# Recent Advances in the Synthesis of Carotenoid-Derived Flavours and Fragrances

**DOI:** 10.3390/molecules200712817

**Published:** 2015-07-15

**Authors:** Stefano Serra

**Affiliations:** C.N.R., Istituto di Chimica del Riconoscimento Molecolare, Via Mancinelli 7, I-20131 Milano, Italy; E-Mail: stefano.serra@cnr.it; Tel.: +39-02-2399-3076; Fax: +39-02-2399-3180

**Keywords:** carotenoids, apocarotenoids, flavours, fragrances, organic synthesis, stereoselective synthesis, resolution, enzymes

## Abstract

Carotenoids are important isoprenoid compounds whose oxidative degradation produces a plethora of smaller derivatives, called apocarotenoids, which possess a range of different chemical structures and biological activities. Among these natural products, compounds having less than 15 carbon atoms in their frameworks are often relevant flavours or fragrances and their manufacturing represents an important economic resource for chemical companies. The strict correlation between stereochemical structure and odour has made the stereospecific synthesis of the latter biological active compounds increasingly important. In this review, the recent advances on the synthesis of the most relevant carotenoid-derived flavours and fragrances are discussed. In particular, the new synthetic methods that have given new and innovative perspectives from a scientific standpoint and the preparative approaches that might possess industrial importance are described thoroughly.

## 1. Introduction

Carotenoids are an important subgroup of isoprenoid compounds, characterized by the presence of a conjugated tetraterpene (C_40_) framework and comprising over 600 chemical structures [1]. They are widely distributed in nature being synthesized [2] by many types of organisms ranging from archae and eubacteria to eukaryotes. The presence of up to 15 conjugated double bonds imparts the pigment character to many carotenoids [3]. These compounds play various important roles in living beings as the pigmentation is important for the life cycle of plants and animals and the ability of absorbing light can impart them the role of photoprotective agents, anti-oxidant compounds and light-harvesting molecules in photosynthetic organisms [1,2,3,4,5].

Carotenoids are vital to many organisms not only in their intact form, but also because they are precursors of a number of other bioactive derivatives which are usually formed by oxidative degradation [1,6,7,8,9]. These compounds are smaller than their precursors and are collectively named apocarotenoids or norisoprenoids, regardless of their specific way of formation.

It has been demonstrated that carotenoid degradation can occur either *via* merely chemical/physical mechanisms or through enzyme(s) catalyzed reactions. Actually, the action of light and of oxygen can effectively oxidize carotenoids to give a number of apocarotenoids, even having the same chemical structure of those obtained by enzyme-mediated oxidation. In addition, the bio-catalyzed degradation processes are in their turn carried out either by non-specific enzymes, including lipoxygenases and peroxidases, or by enzymes with regiospecific cleavage activities on specific double bonds of the carotenoids framework which are called carotenoid cleavage oxygenases (CCOs). This “family of enzymes” comprises both dioxygenases and monooxygenases and their mechanism of action as well as their structural characterization was elucidated only recently. Actually, the latter classes of oxidase differ from one another because of the mechanism of oxygen addition to the substrates. In the monoxygenases, only a single atom of dioxygen is incorporated into a substrate with the other being reduced to a water molecule. The dioxygenases catalyze the oxidation of a substrate without the reduction of one oxygen atom from dioxygen into a water molecule. However, the determination of the mechanism can be ambiguous because many chemical intermediates are usually involved in the reactions. This aspect is significant in carotenoid degradation where different enzyme and chemical intermediates can lead to the same compounds.

Overall, the cleavage products can act as hormones, signaling compounds, chromophores and flavours or fragrances. Actually, the inherent increase of the apocarotenoids’ volatility is often accompanied by the development of a new olfactory activity. This kind of process is quite common especially in vegetal species where the formation of volatiles is part of the life cycle of the plant. As a representative example, we can note that the degradation of carotenoids is responsible of the formation of a number of substances having less than 15 carbon atoms in their frameworks, which characterize the odors of different fruits and flowers during ripening and blossoming, respectively [1,2,3,4,5,6,7,8,9,10]. Among these natural products, the C_13_, C_11_, C_10_, and C_9_ derivatives are the most relevant ones. The combination of the great diversity of the carotenoids chemical structures with the different degradation pathways possible gives rise to a huge number of flavours and fragrances [11].

**Figure 1:**
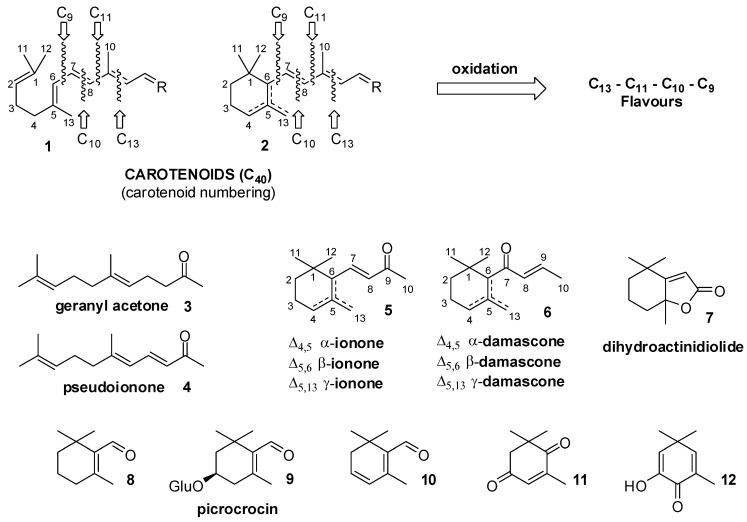
The oxidative cleavage of carotenoids having moiety of type **1** or **2** and some representative examples of the deriving flavours and fragrances.

It is worth noting that the terminal moieties of the carotenoids framework can be either a linear chain or a substituted cyclohexene ring, as graphically described with structures **1** and **2**, respectively (Figure 1). As a result, the oxidation of the compounds possessing a moiety of type **1** affords mainly linear C_13_ flavours as geranyl acetone **3** or pseudoionone **4**, whereas the primary odour constituents derived from carotenoids of type **2** are cyclohexene derivatives such as ionone and damascone isomers **5** and **6**, respectively.

The possibility of three different positions of the cyclohexene double bond, the presence of a stereogenic centre in position 6 (carotenoids numbering) and the eventual structural rearrangements, afford a large number of isomers. Also, the formation of bicyclic derivatives is a common degradation pathway. Concerning C_11_ derivatives, the example of dihydroactinidiolide **7** is representative of a number of bicyclic lactones identified from many natural sources and possessing high chemical stability. Conversely, the C_10_ aldehydes **8**, **9** and **10** are very relevant flavours whose employment as flavour/fragrances is limited by their low stability and high reactivity. β-Cyclocitral **8** is formed by oxidation of β-carotene whereas the glucoside picrocrocin **9** is a degradation product of the carotenoid zeaxanthin glycoside. Compound **9** is an odourless apocarotenoid occurring in saffron, where through the action of the enzyme glycosidase, it liberates the aglycone which is then transformed into safranal **10**. The latter aldehyde, beside a number of other C_9_ apocarotenoids such as the keto-derivatives **11** and **12**, is responsible of the unique flavour of saffron [12].

The relative abundance and diffusion in nature of a given apocarotenoid in turn depend on the abundance of the parent carotenoids as well as on the chemical stability of the apocarotenoid itself. For instance, β-ionone, dihydroactinidiolide and ketoisophorone can be produced by the degradation of different widespread carotenoids and are quite stable compounds. Consequently, the aforementioned compounds are so widely distributed in plants and algae that have been detected in trace amount also in ocean water and have been considered among potential volatile markers for the determination of sea salt origin [13].

A number of other relevant apocarotenoids used as flavours or fragrances have been reported so far. Nevertheless, this review will focus on the synthetic approaches that have affected this field the most, limiting the discussion on the research that has been described in the last 20 years.

## 2. Preparation of “Natural” Carotenoid-Derived Flavours and Fragrances

The origin of flavours also affects their commercial value [14]. The legislations of different states have officially stated that “natural” flavour substances can only be prepared either by physical processes (extraction from natural sources) or by enzymatic or microbial processes, which involve precursors isolated from nature. Although US [15] and EU [16] regulatory requirements have distinct differences, this rough classification has created a dichotomy in the market because compounds labeled as “natural” become profitable products, whereas other flavours that occur in nature but are obtained through chemical methods are less appreciated by the consumer. From a chemical point of view, there is no difference between a compound synthesized in nature and the identical molecule produced in the laboratory, but still the price of a flavour sold as natural is often significantly higher than those prepared by chemical synthesis.

As mentioned above, a number of apocarotenoids are used as flavour ingredients but mostly occur in nature as trace components. Therefore, their extraction is not a viable process and the “natural” routes for their production, as the bioconversions of natural precursors using biocatalysis or the *de novo* synthesis (fermentation), would be of high industrial interest. On the contrary, some carotenoids are readily available by extraction from vegetable (lycopene, carotene) and can be suitable precursors for the preparation of natural apocarotenoids.

In the past, the incomplete knowledge of the mechanisms of enzyme-mediated carotenoids cleavage as well as the difficulties encountered in CCOs’ identification and their structural characterization hampered the developing of enzyme-mediated processes for the production of flavours from carotenoids. Only in recent years, the characterization of a number of carotenoid cleavage dioxygenases (CCDs) from plants allowed the direct preparation of specific flavours from their parent carotenoids. The first work in this field [17] reported the identification of a CCD from *Arabidopsis thaliana*, its overexpression in *Escherichia coli* and the use of the obtained recombinant protein for the cleavage of β-carotene, lutein, and zeaxanthin to produce the corresponding C_13_ ionone derivatives. Afterwards, a large number of CCDs were isolated from plants such as petunia [18], melon [19], maize [20], *Rosa damascena* [21], *Osmanthus fragrans* [22], carrot [23], grape [24], tomato [25] and saffron [26] and then were overexpressed in heterologous hosts. The obtained enzymes were able to cleave different carotenoids affording farnesylacetone, ionone isomers (mainly β and α isomer), pseudoionone, geranylacetone, citral, β-cyclocitral, 6-methyl-5-hepten-2-one and crocetin.

Very recently, a further important advance has been made through the development of a synthetic biology approach for β-ionone production from glucose [27]. The genes responsible for the β-carotene biosynthesis and for CCD production were expressed in *Saccharomyces cerevisiae* from a single polycistronic construct. As a result, glucose-grown cultures of the engineered strain were able to produce β-ionone and geranylacetone.

It is worth noting that the detailed study of carotenoid cleavage enzymes has furnished a powerful tool for the biotechnological production of many flavours and fragrances. Already now, it is possible to engineer a new metabolic pathway in a heterologous host, in order to obtain a given metabolite. Of course, this approach is far from being straightforward and usually the development of an industrial process based on these kinds of biotransformations is a demanding process that takes a long time and much effort. Therefore, it is no surprise that the above described research limited the study on the scientific aspects and completely neglected the preparative issues. A few exceptions are noteworthy. For example, fungi [28] or fungal peroxidases from *Marasmius scorodonius* in combination with hydrogen peroxide [29] were used for the synthesis of volatile apocarotenoids starting from different carotenoids. In addition, a new preparative process for the synthesis of natural β-ionone from β-carotene has been developed [30]. Accordingly, recombinant CCDs and oxygen are able to oxidize β-carotene without the employment of any additional cofactors. The produced β-ionone was separated from the reactor by means of organophilic pervaporation.

## 3. New Synthetic Approaches to the Most Relevant Classes of Apocarotenoids Odorants

As described above, although apocarotenoids have common biosynthetic origin, they possess chemical structures very different from each other. As a consequence, the synthetic issues related to the latter flavours and fragrances as well as the recent advances that have been studied to overcome the same are strictly linked to their specific chemical frameworks. Furthermore, it is important to note that flavours and fragrances are often a complex mixture of isomers, and each one might possess a specific sensory property. It is known that the isomeric composition (regio- and/or stereoisomeric) of an odorous compound greatly affects its olfactory properties either in terms of quality of its perceived odour or in terms of its odour threshold [31]. Consequently, only one regio/stereoisomer, or a balanced combination of them, might be used in aroma, fragrances or cosmetic formulations. Moreover, the preparation of enantioenriched compounds needs of asymmetric synthesis or the use of optical active starting materials, greatly increasing both the complexity of the synthetic approaches and the production cost.

### 3.1. Synthesis of Ionone and Damascone Isomers 

**Figure 2:**
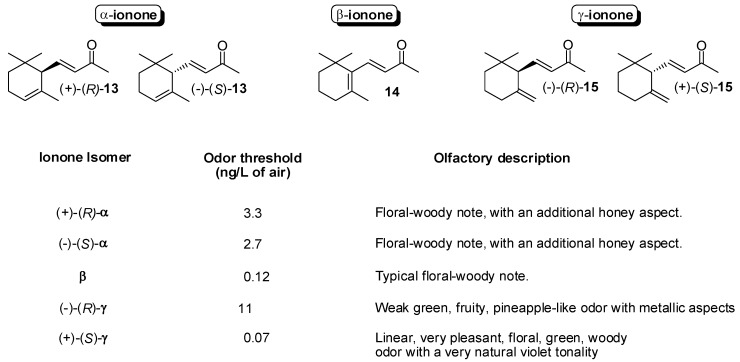
Odor descriptions and detection thresholds of ionone isomers.

Ionones are relevant examples of flavour/fragrance widely used in industry whose isomeric forms show different odour properties. A comprehensive olfactory evaluation of all the isomers of ionone [32] revealed that (*S*)-γ-ionone **15** is the most powerful and pleasant isomer being about 150 time as powerful as (*R*)-enantiomer and having a very natural violet tonality (Figure 2). In addition, remarkable differences among the odour properties of the ionone, dihydroionone and dehydroionone isomers were reported. Pseudoionone **4** is an inexpensive industrial product and its cyclization afford either α-, or β-ionone depending on the kind of acid used to catalyze the reaction [11]. In addition, the less available γ-ionone can be prepared starting from α-isomer by means of photochemical isomerization of the cyclohexene double bond [33]. Hence, racemic ionone isomers have become quite affordable commodities. Conversely, the enantioselective synthesis of the ionone framework is especially demanding and only few approaches have preparative significance. Therefore, the resolution of racemic α-ionone was the first studied process to prepare enantioenriched ionone isomers. The classical optical resolution of α-ionone by fractional crystallization of its menthylhydrazone derivative [34] turned out to be an underperforming process and new approaches, based on enzymatic resolution, have been developed [35].

**Figure 3:**
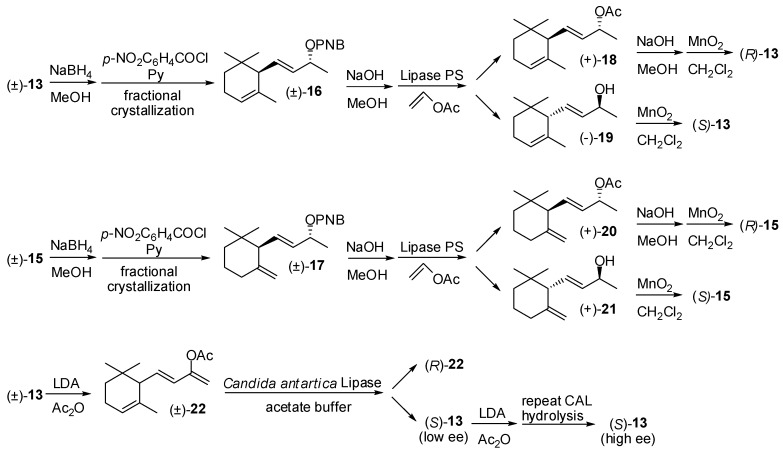
Lipase-mediated enantioselective synthesis of ionone isomers.

Accordingly, it was found out that lipase PS catalyses the acetylation of ionol isomers having (9*R*) absolute configuration with very high enantioselectivity [36,37] (Figure 3). NaBH_4_ reduction of racemic α-ionone **13** or γ-ionone **15** afforded a diastereoisomeric mixture of ionol isomers which were transformed into the corresponding *p*-nitrobenzoate esters. The fractional crystallization of these compounds gave diastereoisomerically pure derivatives **16** and **17**. The alkaline hydrolysis of the latter esters provided the corresponding racemic ionol isomers that were acetylated using vinyl acetate and lipase PS as catalyst. The obtained enantiopure acetate (+)-**18** and (+)-**20** were hydrolyzed using NaOH in methanol and the resulting ionol isomers were then converted into (*R*) enantiomer of α-ionone and γ-ionone, respectively by means of MnO_2_ oxidation. Moreover, the unreacted ionols (−)-**19** and (+)-**21** provided the (*S*) enantiomer of the same ionone isomers.

More recently, researchers from Takasago International [38] have developed a process based on the lipase-mediated hydrolysis of enolacetate **22**, in turn prepared from racemic α-ionone. The hydrolysis of the latter ester using lipase from *Candida antarctica* afforded enantioenriched (*S*)-ionone and the (*R*)-enantiomer of the enol acetate **22**. Although the process can be easily performed on a preparative scale, the enantioselectivity of the key step appear to be modest and the whole process should be repeated twice to obtain α-ionone in high enantiomeric purity.

**Figure 4:**
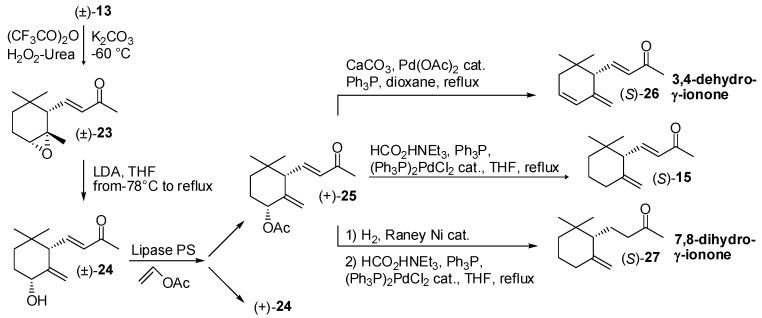
Lipase-mediated resolution of 4-hydroxy-γ-ionone: Enantioselective synthesis of γ-ionone derivatives.

In order to set out a versatile procedure for the preparation of enantioenriched γ-ionone derivatives, the enantio- and diastereoselective lipase-mediated acetylation of 4-hydroxy-γ-ionone **24** (Figure 4) was successfully exploited [39,40]. The latter ionone derivative is easily preparable through base-mediated transposition of epoxy-α-ionone **23**, in turn obtained by diastereoselective epoxidation of α-ionone. Lipase PS catalyzed the acetylation of (4*R*,6*S*)-4-hydroxy-γ-ionone with complete diastereoselectivity and high enantioselectivity. The obtained acetate (+)-**25** was transformed into the (*S*)-3,4-dehydro-γ-ionone **26** by palladium-catalyzed elimination of the acetate functional group. In the same way, the regioselective reduction of the latter allyl ester using triethylammonium formate and (Ph_3_P)_2_PdCl_2_ catalyst provided a reliable synthetic path either to (*S*)-γ-ionone **15** or (*S*)-7,8-dihydro-γ-ionone **27**. It is worth noting that the latter compound is a relevant fragrance component of ambergris absolute and both enantioenriched acetate **25** and ketone (*S*)-**27** have been used as chiral building blocks for the synthesis of a number of natural products [41,42,43,44].

Epoxy-α-ionone as well as 4-hydroxy-β-ionone and 4-hydroxy-β-damascone occur in nature in enantioenriched form. Although these compounds are not olfactory active compounds in their own right, it is thought they are precursors of other flavours.

**Figure 5:**
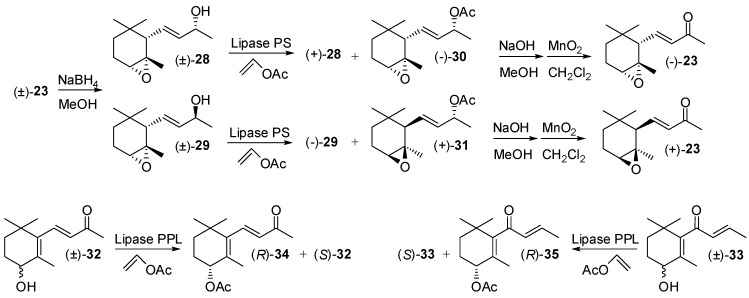
Lipase-mediated resolution of epoxy-ionone, 4-hydroxy-ionone and 4-hydroxy-damascone isomers.

The reduction of epoxy-α-ionone **23** with NaBH_4_ gives a mixture of the racemic epoxy-ionols **28** and **29** that can be separated through chromatography. Each one of the diastereoisomeric alcohols was resolved using lipase PS-mediated acetylation [45]. The obtained enantioenriched acetates (−)-**30** and (+)-**31** that can be easily transformed in the corresponding epoxy-α-ionone enantiomers (Figure 5). Similarly, racemic 4-hydroxy-β-ionone and 4-hydroxy-β-damascone were resolved through pig pancreatic lipase catalyzed *trans*-esterification to afford enantioenriched acetate (*R*)-**34**, (*R*)-**35** [46] and alcohol (*S*)-**32**, (*S*)-**33**, respectively.

**Figure 6:**
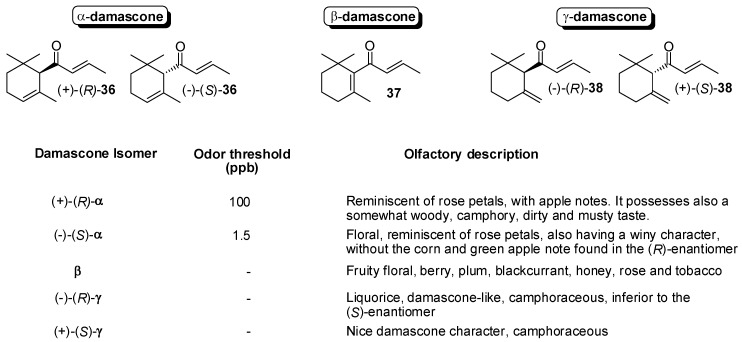
Odor descriptions and detection thresholds of damascone isomers.

Due to the steric crowding around the position 7 of damascone framework (Figure 1 and Figure 6), the esterification or the hydrolysis of the hydroxyl/ester functional groups linked to C(7) is not catalyzed by the most common hydrolytic enzymes. As a consequence, the reported enantioselective syntheses of damascone isomers were not based on the enzyme-mediated resolution approaches.

**Figure 7:**
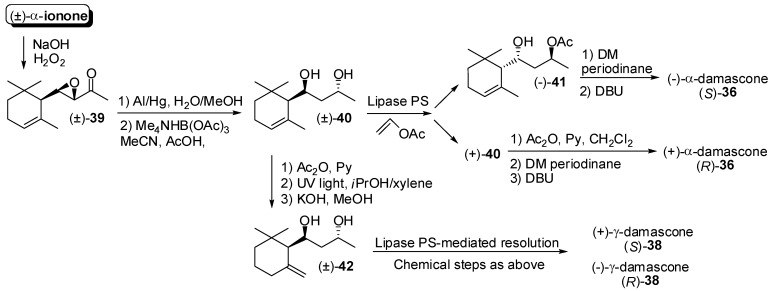
Lipase-mediated enantioselective synthesis of α- and γ-damascone enantiomers.

A significant exception, involving the resolution of 7,9-dihydroxy derivatives, has been recently reported [47]. Accordingly, epoxidation of the conjugated double bond of racemic α-ionone (Figure 7) using hydrogen peroxide in presence of NaOH afforded ketone **39** with complete regio- and diastereoselectivity. The regioselective reductive opening of the epoxide ring using aluminum amalgam followed by the diastereoselective reduction of the ketone functional group gave diastereoisomerically pure diol **40**. The latter racemic compound was resolved according to the lipase PS-mediated acetylation process described for ionone isomers. In fact, the hydroxyl group in position 7 of the ionone framework did not react and only 9-acetyl derivative was formed. Afterward, the enantiopure hydroxyl-acetate (−)-**41** was oxidized using Dess-Martin periodinane and the following reaction with DBU allowed the elimination of the acetate functional group affording (*S*)-α-damascone **36**. Similarly, enantioenriched diol (+)-**40** was chemically transformed into hydroxy-acetate (+)-**41** which was converted into (*R*)-α-damascone as described above. It is worth noting that diol **40** was also transformed into diol **42** by photochemical isomerization. The application of the above described process allowed the transformation of racemic **42** into enantioenriched (*S*)- and (*R*)-γ-damascone **38**.

**Figure 8:**
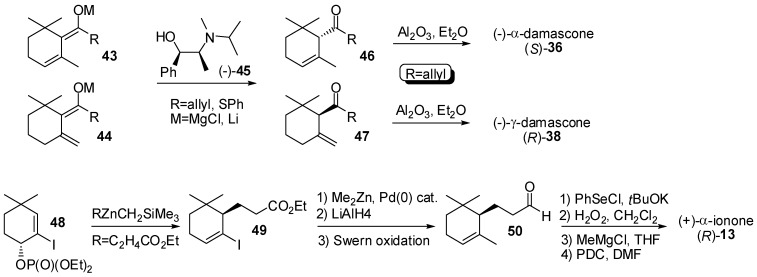
Asymmetric syntheses of ionone and damascone isomers.

**Figure 9:**
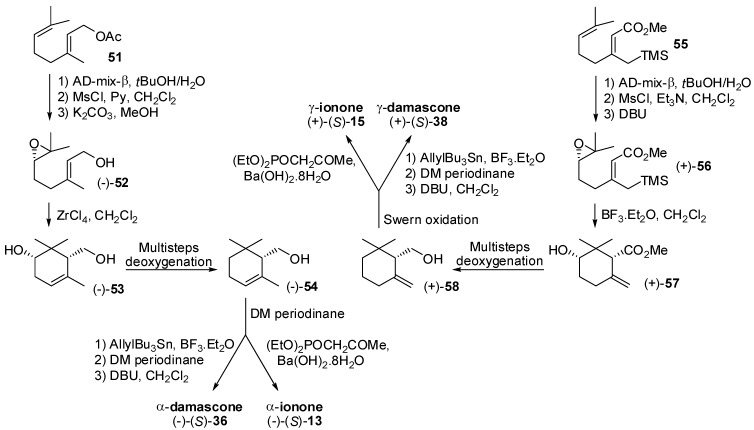
Asymmetric syntheses of ionone and damascone isomers.

**Figure 10:**
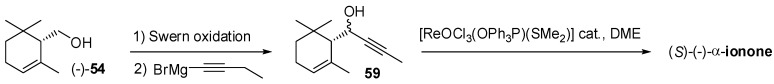
Asymmetric synthesis of (*S*)-α-ionone through Meyer-Schuster rearrangement.

Beside the described resolution-based processes, different asymmetric synthesis of ionone and damascone isomers have been reported (Figure 8, Figure 9 and Figure 10).

During the late 80s, it was discovered that the enantioselective protonation of enolates of type **43** and **44** (Figure 8) with ephedrine derivatives such as the compound (−)-**45** affords enantioenriched ketones of type **46** and **47**, respectively. The process was successfully exploited for the preparation of both enantioforms of α- and γ-damascone [48,49,50]. For example, enolates of type **43** and **44** having the allyl group as substituent were protonated using (−)-**45** as a proton source and the resulting β,γ-unsaturated ketones **46** and **47** were isomerized using Al_2_O_3_ to give (*S*)-α- and (*R*)-γ-damascone, respectively, in high enantiomeric purity. As the conversion of the damascone isomers in the corresponding ionone isomers is possible, an indirect enantioselective preparation of the latter compounds was also assured [51]. More recently, (*R*)-α-ionone **13** was prepared starting from enantiopure allylic phosphate **48** [52]. The stereocenter in position 6 of the ionone framework was built up through a copper-catalyzed, highly stereoselective S_N_2ʹ allylic substitution of the phosphate ester with a mixed diorganozinc derivative. The obtained ester **49** was then transformed into enantiopure ketone **13** by a number of functional groups interconversion comprising of the substitution of the iodo atom with the methyl group, the formation of the 7,8-(*E*) double bond and the stepwise transformation of the ethyl ester group into the methylketone group.

Conversely, the asymmetric synthesis of the cyclohexene moiety of the ionone and damascone isomers were accomplished through the stereoselective cyclization of enantioenriched epoxy-derivatives of type **52** and **56** (Figure 9), which were in turn synthesized by means of Sharpless asymmetric dihydroxylation as the key step. The ZrCl_4_-promoted cyclization of enantiopure geraniol epoxide (−)-**52** gave diol (−)-**53** which was used to prepare α-cyclogeraniol (−)-**54**. The latter alcohol was oxidized and the obtained aldehyde was used for the synthesis of (*S*)-α-ionone and (*S*)-α-damascone [53].

Similarly, the BF_3_-catalyzed cyclization of epoxyallylsilane (+)-**56** gave hydroxy-ester (+)-**57** [54] that possess the exomethylenic double bond necessary for the synthesis of γ-ionone/damascone isomers. Accordingly, (+)-**57** was transformed into γ-cyclogeraniol (+)-**58** which was used for the synthesis of (*S*)-γ-ionone **15** and (*S*)-γ-damascone **38**. Furthermore, enantiopure alcohol (−)-**54** was used as building block for a new stereoselective synthesis of (*S*)-α-ionone **13** based on the Re-catalyzed Meyer-Schuster rearrangement [55] of the propargylic alcohol **59** (Figure 10).

**Figure 11:**
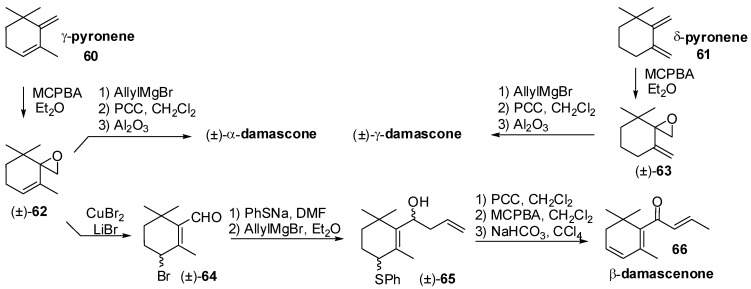
The synthesis of racemic α- and γ-damascone isomers and of β-damascenone starting from γ- and δ-pyronene.

As a final point, it is worth noting that a new synthetic entry to racemic α-damascone, γ-damascone and β-damascenone has been described [56]. Accordingly, γ-pyronene **60** and δ-pyronene **61**, were regioselectively epoxidized to give epoxides **62** and **63**, respectively (Figure 11). The sequential treatment with allyl magnesium bromide, oxidation of the resulting carbinols and base-catalyzed isomerization gave α-damascone and γ-damascone, respectively. The treatment of epoxide **62** with LiBr in presence of CuBr afforded aldehyde **64** that was transformed into C_13_ derivative **65** through stepwise substitution of the bromine atom with the thiophenyl group and addition of allylmagnesium bromide to the aldehyde group. The obtained secondary alcohol was then transformed into β-damascenone **66** by a number of functional groups interconversion comprising of the oxidation of hydroxy and thiophenyl groups and elimination of the resulting phenylsulfonyl group.

### 3.2. Synthesis of Other Relevant C_13_ Apocarotenoids

**Figure 12:**
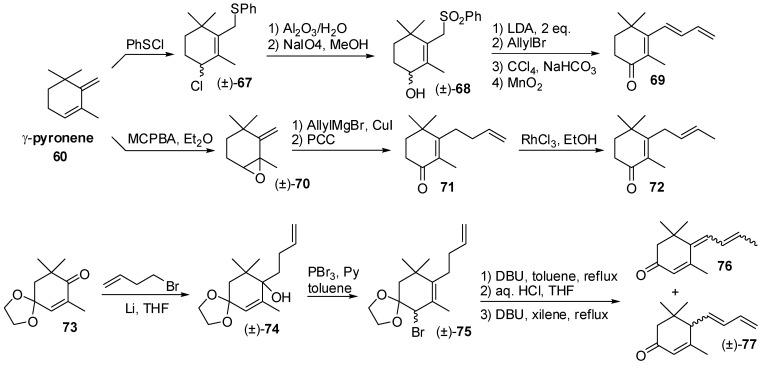
The regioselective synthesis of megastigma-4-one **69** and **72** and of megastigma-3-one isomers **76** and **77**.

Although ionone and damascone isomers have pivotal relevance in flavour and fragrance field, different synthetic approaches to other C_13_ apocarotenoids are worth mentioning. For example, megastigmatrienone and megastigmadienone isomers are constituents of the aroma of different fruits as well as they are present in rums and tobacco. Two regioselective syntheses of megastigma-4-one [57] and of megastigma-3-one [58] isomers have been developed (Figure 12). The reaction of γ-pyronene **60** with phenylsulfenyl chloride gave derivative **67**. The substitution of the chloride atom with a hydroxy group and the oxidation of the thiophenyl group to the corresponding phenylsulfonyl group gave derivative **68** that was alkylated with allyl bromide followed by elimination of the phenylsulfonyl group. Finally, the oxidation of the obtained trienic alcohol afforded megastigma-5,7,9-trien-4-one **69**.

Conversely, copper catalyzed addition of allyl magnesium chloride to compound **70** gave megastigma-5,9-dien-4-ol which was oxidized to the corresponding megastigma-5,9-dien-4-one **71** whose monosubstituted double bond can be isomerized using RhCl_3_ as catalyst to afford megastigma-5,8-dien-4-one **72**.

A general approach to the megastigma-3-one isomers synthesis has been achieved using conjugate dehydrobromination of allylic bromide of type **75** as the key step. Accordingly, treatment of oxophorone monoethylene ketal with a suitable C_4_ organo-lithium derivative gave carbinol **74** that was converted into bromide **75** by reaction with PBr_3_ and pyridine. The dehydrobromination of **75** with DBU followed by acid hydrolysis of the ketal functional group and DBU-catalyzed isomerization of the double bond(s) afforded megastigma-4,6,8-trien-3-one **76** and megastigma-4,7,9-trien-3-one **77**.

**Figure 13:**
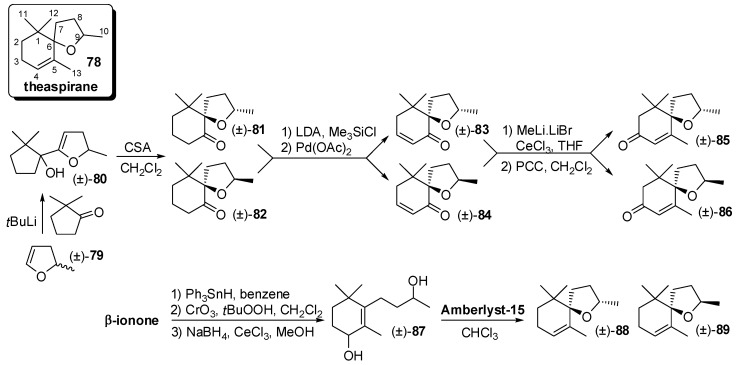
Syntheses of odorants having the generic theaspirane framework: Racemic theaspirone and theaspirane isomers.

Compounds having the general theaspirane framework **78** (Figure 13) represent a further relevant class of carotenoid-derived flavours and fragrances. Theaspirane is in its own right an appreciate flavour ingredient and occurs in a minute amount in various essential oils such as raspberry oil and passionfruit oil. Other derivatives such as vitispirane are present in fruit and one isomer of the 3-oxo-theaspirane, namely *cis*-theaspirone **86** is a key flavour of the black tea.

Racemic theaspirones isomers were prepared from 2,2-dimethylcyclopentanone [59]. The reaction sequence began with 1,2-addition of the lithium derivative of 2-methyl-2,3-dihydrofuran **79** to this ketone and immediate acid-catalyzed ring expansion of the resulting carbinols **80** to give a separable pair of spiro ethers **81** and **82**. Individual conversion of these diastereoisomers to unsaturated ketones **83** and **84**, respectively, is followed by tandem condensation with the methyllithium-lithium bromide complex and oxidation with pyridinium chlorochromate to afford theaspirones **85** and **86**, respectively.

Furthermore, it is important to note that the construction of the spiroether framework by acid-catalyzed cyclization has been subjected of a number of studies. In particular, it was discovered that the ion-exchange resin amberlyst-15 catalyses the oxaspirocyclisation of racemic diol of type **87** affording theaspirane isomers **88** and **89** in almost quantitative yield [60].

**Figure 14:**
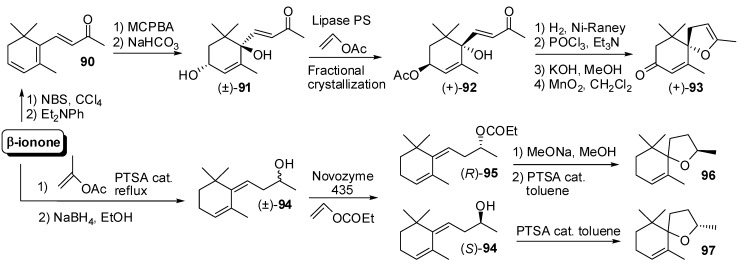
Enantioselective syntheses of odorants having the generic theaspirane framework through the resolution approach: 7,8-Dehydrotheaspirone and theaspirane isomers.

The 8,9-dehydrotheaspirone **93** (Figure 14) was identified in different natural source such as tobacco, nectarines, Riesling wine and honey. The diastereoisomerically pure diol **91** was prepared starting from β-ionone and then submitted to the lipase PS-mediated acetylation [61]. The fractional crystallization of the obtained acetate (+)-**92** allows increasing efficiently its enantiomeric purity. The conjugated double bond of the resulting enantiopure compound **92** was regioselectively hydrogenated using Ni-Raney to give an intermediate bicyclic hemiketal whose dehydratation, followed by transformation of the acetate functional group into the ketone functional group, afforded enantiopure dehydrotheaspirone (+)-**93**.

Similarly, racemic alcohol **94** was prepared from β-ionone and then was resolved by means of NOVOZYME 435-mediated esterification [62] using vinyl propionate as acyl donor. The obtained enantiopure propionate (*R*)-**95** was hydrolyzed and the resulting alcohol (*R*)-**94** was cyclised by heating in toluene in presence of catalytic PTSA to give (*R*)-theaspirane **96**. It is worth noting that compound **96** consisted of an equimolar mixture of enantiopure diastereoisomers possessing strong fruity flavour whereas the theaspirane isomers mixture (*S*)-**97** obtainable starting from the unreacted alcohol, namely (*S*)-**94**, had camphor-like, woody-like odour.

**Figure 15:**
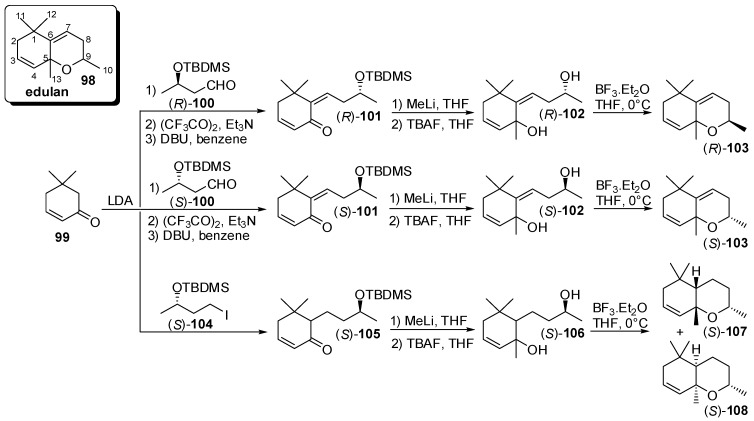
Enantioselective synthesis of odorants having the generic edulan framework: Edulan and 6,7-dihydroedulan isomers.

Further relevant apocarotenoid characterized by a bicyclic ethers structure are those possessing the generic edulan framework **98** (Figure 15). Edulan and 6,7-dihydroedulane isomers exhibits intense rose-like aromas and are important trace components of the flavour of the purple passionfruit (*Passiflora edulis*) whereas 3,4-dihydro-3-oxo-edulan isomers possess tobacco-like odour and were found in tobacco, passionfruit, oak wood and Chardonnay juice.

As described for theaspiranes, the reported stereoselective syntheses of edulans are based on two steps: the stereocontrolled formation of at least one of the two stereocenter placed next to the oxygen atom (usually the secondary one) and the formation of the pyran ring by cyclization. Accordingly, a work dedicated to the assignation of the absolute configuration to these compounds [63] described the preparation of the edulan isomers through the alkylation of cyclohexanone **99** with the chiral building blocks (*R*)- and (*S*)-**100**, in turn obtained from (*R*) and (*S*)-3-hydroxybutyric acid methyl ester. After elimination reaction, the obtained C_13_ derivatives (*R*)- and (*S*)-**101** were transformed into the diols (*R*)- and (*S*)-**102** which were cyclised using boron trifluoride etherate to afford edulans (*R*)- and (*S*)-**103**, each one made up of two enantiopure diastereoisomers.

Similarly, the alkylation of cyclohexanone **99** with iodide (*S*)-**104** provided the C_13_ derivative (*S*)-**105** which was transformed into diol (*S*)-**106**. In principle, the cyclization of the latter compound could afford up to four diastereoisomers but only 6,7-dihydroedulan isomers (*S*)-**107** and (*S*)-**108** were obtained, most likely because of the preferred formation of the tetrahydropyranyl ether with *cis* ring junction.

**Figure 16:**
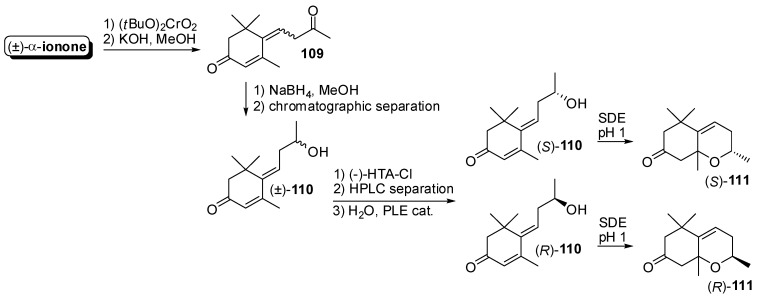
Enantioselective synthesis of odorants having the generic edulan framework through the resolution approach: 3,4-Dihydro-3-oxo-edulan isomers.

An analogous synthetic approach has been developed in order to study the chiral composition of natural 3,4-dihydro-3-oxo-edulan isomers [64] (Figure 16). For that reason, racemic 3-oxo-*retro*-ionol **110** was prepared by reduction of the 3-oxo-*retro*-ionone **109**, which were in turn obtained starting from racemic α-ionone. Alcohol **110** was then transformed in the corresponding (*R*)-2-phenylpropionate derivative and the resulting diastereoisomeric esters were separated through preparative HPLC and then were hydrolyzed using PLE to give enantiopure alcohols (*S*)-**110** and (*R*)-**110**. The latter compounds were subjected to simultaneous distillation/extraction treatment (SDE) at pH 1 and afforded 3,4-dihydro-3-oxo-edulan (*S*)-**111** and (*R*)-**111**, respectively, each one made up of two enantiopure diastereoisomers.

### 3.3. Synthesis of Relevant Apocarotenoids Having a C_11_ Chemical Framework

Even though the family of the C_13_ apocarotenoids comprises a large number of structurally different components, many of which are especially sought after for their flavour and fragrance properties, further smaller carotenoid-derived compounds have drawn both the industrial and academic interest.

**Figure 17:**
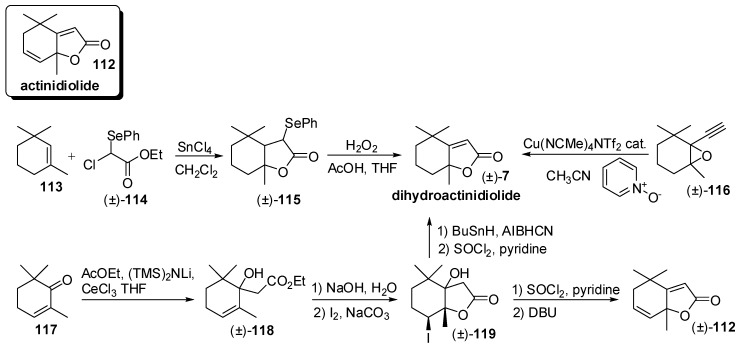
Syntheses of odorants having the generic actinidiolide framework: Racemic actinidiolide and dihydroactinidiolide.

**Figure 18:**
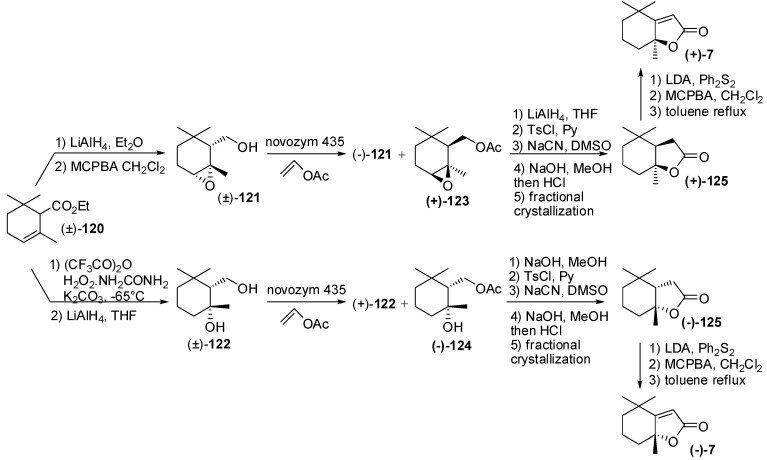
Enantioselective synthesis of odorants having the generic actinidiolide framework through the resolution approach: Tetrahydroactinidiolide and dihydroactinidiolide.

Among these compounds, the bicyclic C_11_ lactones possessing the general actinidiolide framework **112** (Figure 17) are widespread in nature and comprise, together with compounds **112**, the dihydroactinidiolide **7** and the corresponding tetrahydro derivatives **125** (Figure 18), a number of further hydroxylated derivatives. Although actinidiolide **112** has been isolated only from the essential oil of *Actinidia polygama*, the structurally related dihydroactinidiolide and *cis*-tetrahydroactinidiolide have been recognized as flavour components in many natural sources. More specifically, these compounds were identified in a number of vegetal or in their derivatives such as tea, tobacco, wine, apricot, algae, melon and even cactaceae. Due to their biological properties and to their intriguing chemical structure, these lactones were synthesized mainly in order to furnish their comprehensive chemical characterization as well as to prove the effectiveness of the new proposed synthetic methods.

Recently, different preparative approaches have been reported. The reaction of 1,3,3-trimethylcyclohexene **113** with α-chloro-α-phenylseleno ethyl acetate **114** afforded lactone **115** [65]. The latter compound was oxidized to the corresponding selenoxide which eliminated swiftly to afford racemic dihydroactinidiolide **7**. This lactone was also efficiently prepared by oxidative cyclization of the alkynyl oxirane **116** using the combination of pyridine oxide and Cu(NCMe)_4_NTf_2_ catalyst [66]. Another effective method was based on the selective 1,2-addition of the cerium enolate of ethyl acetate to 2,6,6-trimethylcyclohexenone **117** [67]. The ester group of the obtained adduct **118** was hydrolyzed and the resulting acid was converted into lactone **119** by means of iodolactonization reaction. Reduction of the iodide functional group followed by elimination of the tertiary hydroxyl group gave dihydroactinidiolide **7** whereas elimination of both functional groups afforded actinidiolide **112**.

These lactones were also prepared in enantioenriched form either by enzyme mediated resolution or by asymmetric synthesis (Figure 18). *cis*-α-Epoxy-cyclogeraniol **121** and *cis*-5-hydroxy-cyclogeraniol **122** were synthesized diastereoselectively starting from easy available α-cyclogeraniate **120** [68] and the following NOVOZYM 435 catalyzed acetylation provide enantioenriched esters (+)-**123** and (−)-**124**, respectively. The latter acetates were transformed into tetrahydroactinidiolide enantiomers (+)-**125** and (−)-**125**, whose optical purity were efficiently increased by fractional crystallization. The obtained enantiopure lactones were converted into the corresponding dihydroactinidiolide enantiomers through a well-known two step process consisting of alkylation with diphenyldiselenide and oxidative elimination of the obtained α-phenylselenyl derivatives. Since both tetrahydroactinidiolide and dihydroactinidiolide are natural flavours that might be used in foods and beverage, a further selenium-free procedure for the transformation of tetrahydroactinidiolide into dihydroactinidiolide was described.

Accordingly, the use of diphenyldisulfide instead of diphenyldiselenide followed by MCPBA oxidation and thermal elimination of the obtained sulfone proved to be an efficient and reliable method for the transformation of tetrahydroactinidiolide into dihydroactinidiolide.

**Figure 19:**
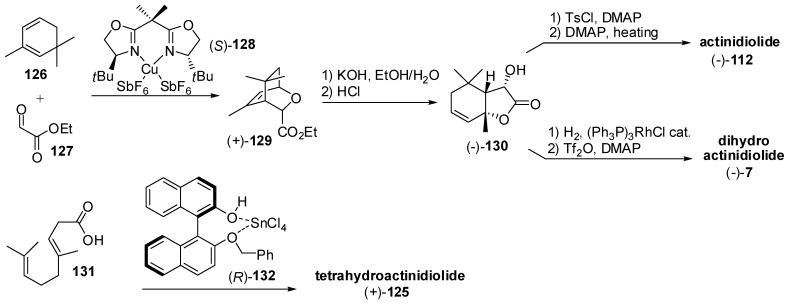
Asymmetric syntheses of odorants having the generic actinidiolide framework: Actinidiolide, dihydroactinidiolide and tetrahydroactinidiolide.

Lastly, two new asymmetric approaches to actinidiolide derivatives have been developed (Figure 19). The chiral copper(II)-bisoxazoline complex (*S*)-**128** was used to catalyse the Diels-Alder reaction of 2,6,6-trimethyl-1,3-cyclohexadiene **126** with ethyl glyoxylate **127** [69]. The obtained enantiopure bicyclic derivative (+)-**129** was transformed into lactone (−)-**130** through the hydrolysis of the ethyl ester followed by acid-catalyzed rearrangement. The latter compound was subjected to either elimination reaction of the hydroxyl functional group or to double bond hydrogenation followed by elimination of the hydroxyl group, to give actinidiolide (−)-**112** or dihydroactinidiolide (−)-**7**, respectively. Furthermore, the (*R*)-benzyl-BINOL SnCl_4_ complex **132** catalyzed the enantioselective cyclization of homogeranic acid **131** to give (+)-tetrahydroactinidiolide **125** in good enantiomeric purity [70].

### 3.4. Synthesis of Odour Active Apocarotenoids Having Less Common Chemical Frameworks 

As mentioned in the introduction, the carotenoids breakdown could involve complex structural rearrangements as well as extensive fragmentation even to give very small derivatives. As a result, some apocarotenoids having less common skeletons were identified in nature and then were synthesized to evaluate their olfactory properties.

**Figure 20:**
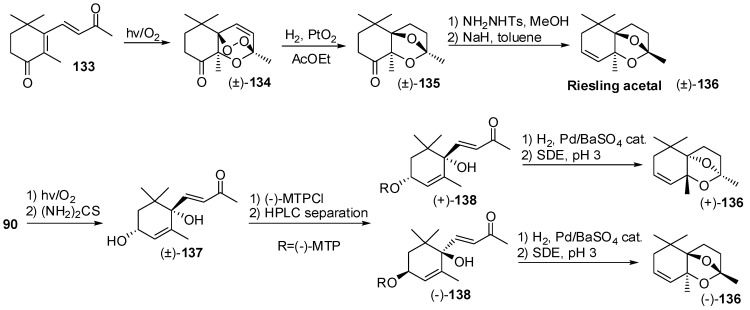
Odorants having less common chemical framework: Synthesis of Riesling acetal in racemic and enantioenriched form.

For example, the so-called Riesling acetal **136** (Figure 20) was identified in Riesling wine and quince brandy. A recent synthesis of the racemic form of this compound was accomplished by means of catalytic hydrogenation of peroxide **134** as the key step [71]. The latter compound is easily available by photochemical reaction of 4-keto-β-ionone with oxygen and its reduction with hydrogen in presence of Pt(0) catalyst gave ketal derivative **135**. Finally, the transformation of the ketone functional group of **135** into the corresponding to sylhydrazone derivative followed by Bamford-Stevens reaction afforded Riesling acetal **136**.

Otherwise, the photochemical reaction of 3,4-dehydro-β-ionone **90** with oxygen and the reduction of the formed bicyclic peroxide using thiourea give racemic diol **137** as a single diastereoisomer. The secondary alcohol group of the latter compound was transformed into the corresponding (−)-MTPA ester and the obtained enantiopure diastereoisomers were separated by preparative HPLC [72]. The regioselective hydrogenation of the conjugated double bond of the latter esters give the corresponding intermediate bicyclic hemiketals whose thermal treatment by simultaneous distillation/extraction treatment at pH 3 afforded (+)- and (−)-Riesling acetal isomers that differed distinctly in their odour properties.

**Figure 21:**
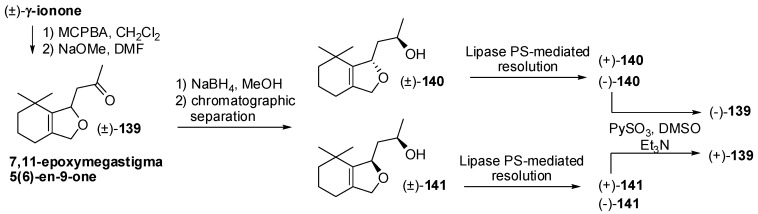
Odorants having less common chemical framework: Enantioselective synthesis of 7,11-epoxymegastigma-5(6)-en-9-one and 7,11-epoxymegastigma-5(6)-en-9-ol isomers.

7,11-Epoxymegastigma-5(6)-en-9-one **139** is a further less common apocarotenoid that was found in passionfruit and in the scent of different orchid flowers where occurs together with diastereoisomerically alcohols **140** and **141** (Figure 21). The epoxidation of racemic γ-ionone with MCPBA followed by treatment with NaOMe afforded bicyclic ketone **139**. The latter compound was reduced using NaBH_4_ in methanol to give alcohols **140** and **141** that were separated by chromatography and were submitted to enzyme-mediated resolution process using lipase PS as catalyst [73]. The obtained enantioenriched alcohols were also oxidized to provide ketone **139** enantiomers, allowing the olfactory evaluation of its enantiomeric forms as well as of those of the diastereoisomeric alcohols **140** and **141**.

**Figure 22:**
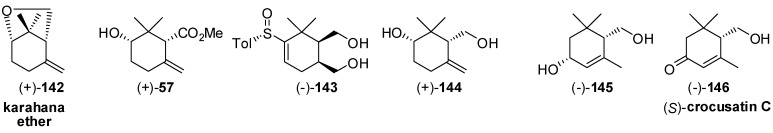
Odorants having less common chemical framework: Enantioselective synthesis of karahana ether and crocusatin C.

Lastly, we report the case of karahana ether (+)-**142** (Figure 22) that is a volatile C_10_ compound, possible deriving by carotenoids degradation, which was isolated from Japanese hops and possesses a pleasant camphor-like odor. Both the enantiomeric forms of this compound were prepared either starting from enantiopure hydroxy-ester (+)-**57** [54], sulfoxide (−)-**143** [74] or diol (+)-**144** [75], which were in turn obtained by stereoselective cyclization of an epoxyallylsilane, by stereoselective Diels-Alder reaction of an enantiopure sulfinyl diene with maleic anhydride or by lipase PS mediated resolution of the racemic diol **144**, respectively. Interestingly, also the isomeric racemic diol **145** was resolved using the same lipase and the enantiopure (3*R*,6*S*) diol (−)-**145** was oxidized to give (*S*)-crocusatin C (−)-**146** [75], which is an aroma component of saffron (*Crocus sativus*).

## 4. Conclusions

This review provides the reader with an overview of the synthetic methods finalized to the study and preparation of carotenoid-derived flavours and fragrances that appeared in the academic and patent literature in the last 20 years. Although a number of heterogeneous chemical structures and synthetic approaches have been presented, a common point can be identified: the growing interest in the preparation and characterization of the odorants in their isomerically pure form. This general trend has already permitted the identification of the chemical compounds that could give the best performance in the preparation of new flavour and fragrance formulations or that could help a better performing of natural flavour reconstruction. Of course, this research is far from being completed and most likely this process will continue in the next years. Due to the interdisciplinary character of this field, the synthetic studies should be supported by the sensorial analysis provided by expert evaluators whereas the production of flavours and fragrances should take advantage of the new tools offered by biocatalysis and of modern methods of asymmetric synthesis.
